# Retired contact sports athletes with cognitive concerns: promoting lifelong brain health

**DOI:** 10.1136/pn-2025-004786

**Published:** 2025-10-08

**Authors:** Neil Graham, Martina Del Giovane, Jessica Hain, Erin Rooney, Karl Zimmerman, Ying Lee, Daniel Friedland, Thomas D Parker, Simon Fleminger, Maneesh C Patel, Richard Sylvester, David Sharp

**Affiliations:** 1 Brain Sciences, Imperial College London, London, UK; 2 UK Dementia Research Institute Centre for Care Research and Technology, Imperial College London, London, England, UK; 3 Imperial College Healthcare NHS Trust, London, UK; 4 Department of Imaging, Imperial College Healthcare NHS Trust, London, UK; 5 Acute Stroke and Brain Injury Unit, National Hospital for Neurology and Neurosurgery, London, UK; 6 Institute of Sport, Exercise and Health, University College London, London, UK

**Keywords:** TRAUMA, PSYCHOL SEQUE, HEAD INJURY, DEMENTIA, CLINICAL NEUROLOGY

## Abstract

There is widespread concern among former athletes about the link between head injury and dementia. Neurologists are increasingly assessing ex-contact sports athletes with cognitive and behavioural issues following repetitive head impacts and traumatic brain injury. Their assessment and management can be challenging due to the broad differential diagnosis, including psychiatric issues, trauma-related impairment and, in some cases, neurodegeneration. There may be a range of pathologies present after trauma exposure, including Alzheimer’s disease and chronic traumatic encephalopathy. Currently, we have only limited understanding of specific clinical phenotypes for distinct types of post-traumatic dementia, nor are there in vivo tests for many of the pathologies. Informed by our experience running a midlife brain health clinic for retired elite contact sport athletes, we describe a practical framework for the workup of athletes with cognitive concerns, highlighting key clinical features, an approach to investigation including neuroimaging and advanced fluid biomarkers, symptomatic management strategies and research directions.

## Introduction

 The long-term effects of traumatic brain injury (TBI) and repetitive blows to the head (‘head impacts’) are of substantial media and societal interest. Chronic traumatic encephalopathy (CTE), a trauma-specific tauopathy,^1^ has been reported in high-profile brain bank case series of American footballers.^2^ CTE has also been reported in smaller post-mortem series including former soccer and rugby players.^3^ Several rugby players in the UK have been diagnosed with young onset dementia during life, which has been attributed to CTE, and an associated class action legal claim is underway.

An important task for the neurologist in this setting is to disentangle the direct effects of TBI, which may be lifelong and fluctuating, from the effects of progressive neurodegeneration that could produce dementia and from important mimics such as mood disorders and/or functional cognitive disorder. While TBI is associated with a higher risk of progressive problems, the presence of TBI is not synonymous with the presence of a progressive neurodegenerative disease.

TBI affects around 50–60 million people each year,^4^ and moderate to severe injuries in particular are a common cause of long-term disability. Patients commonly attend neurology clinics with long-term consequences that are difficult to assess clinically.^5^ The cognitive, psychiatric and functional consequences of TBI are highly variable. Most people make a complete recovery after mild TBI, yet a significant subset may experience slow-to-resolve or persistent symptoms. For example, a recent analysis of the CENTRE-TBI cohort found incomplete recovery in 47% of patients with mild TBI at 6 months. Post-injury trajectories may be highly dynamic, with a subset of patients showing clinical decline years after injury.^6 7^ Interestingly, this is not limited to severe TBI: in the large TRACK-TBI LONG study (n>2000), chronic functional decline occurred in 29% of people after mild TBI, and 26% of those with moderate to severe TBI over the first 7 years.^8^ The causes of these long-term problems are complex, with post-injury symptoms often at the interface of neurology and psychiatry.^9 10^

Former elite/professional male rugby players, who have a substantial history of repetitive head impacts, are at increased risk of dementia, with neurodegenerative disease being around 1.6 to 2.7 times more common.^11 12^ The risk of neurodegenerative disease is also elevated in former soccer players, by an estimated 3.5 times (predominantly dementia).^13^ Distinct from repetitive head impacts, both single and repeated TBIs are associated with dementia: a recent meta-analysis shows a 70% risk increase considering all injury severities.^14^ Very large cohorts provide evidence of a dose–response relationship, with risk greater after severe injury but still present with mild TBI (Danish Civil Registration Study^15^; n=2.8 million, RR_severe_=1.35, RR_mild_=1.17) and multiple TBIs associated with greater risk than single injuries (Swedish Population Study,^16^ n=3.3 million; OR_single mild TBI_ =1.6, OR_single severe_ =2.1, OR_multiple TBI_=2.8). A range of underlying diseases has been reported, including Alzheimer’s disease (AD),^17^ Parkinson’s disease^18^ and motor neurone disease.^19^ CTE is relatively specific to repetitive head impacts,^20^ but former contact sport athletes may also show a range of other neuropathologies, including alpha synuclein, TAR DNA-binding protein 43 (TDP-43), other tauopathies and amyloid-β (Aβ).^21^,^23^ The head impact and biomechanical exposure necessary to produce problems remains unknown, and it is also not known who might be more susceptible or how trauma exposure interacts with prior neurodegenerative or brain ageing trajectories.

In 2021, we established the Advanced Brain Health Clinic (ABHC) at the Institute of Sport, Exercise and Health with an aligned research programme to assess retired professional association footballers and rugby players with concerns about their brain health. To date, we have assessed around 500 retired elite rugby and football (soccer) players. Building on this clinical experience, we describe common presentations, discuss the underlying pathophysiology of chronic TBI and provide a pragmatic approach to the clinical evaluation of the long-term effects of repetitive TBI. Table 1 shows a short case series of patients attending our clinics to illustrate our approach and some of the challenges in this context.

**Table 1:** Illustrative clinical cases of former athletes presenting with cognitive/behavioural concerns

Vignette	Neuropsychology and psychiatry	Neuroimaging	CSF	Consensus diagnosis	Management
Case 1: Patient in their 50s; former professional rugby forward with subjective organisational difficulties managing work schedule; wife notes mild memory/word-finding issues. There was no functional decline.	Performance below expectations on auditory and immediate memory tests. No significant mood or anxiety symptoms were reported.	Clinical MRI: cavum septum pellucidum with perforation; mild generalised sulcal prominence, possibly indicating early volume loss. Research DTI: Normal.	Normal Aβ 42:40 ratio, p-tau181, total tau, NfL, S100B.	Subjective cognitive decline in context of RHI exposure. No objective evidence of neurodegenerative disease.	Advised on optimising lifestyle measures (eg, sleep) to improve performance and longer-term brain health. Scheduled for follow-up.
Case 2: Patient in their 70s; formerly professional association football player (defender, ~20-year career). Known hypercholesterolaemia. Presents with memory difficulties reported by self and family, without functional impairment.	Severe impairment across memory domains (auditory, immediate, delayed recall) >1.5 SD below norms. Executive functions relatively preserved. No significant psychiatric symptoms.	Clinical MRI: Left greater than right temporal lobe atrophy, particularly hippocampus. Research DTI: Normal. Research Amyloid PET: positive.	Low Aβ 42:40 ratio, elevated p-tau181. NfL mildly elevated.	Cognitive impairment due to Alzheimer’s disease.	Started donepezil for symptomatic treatment. Counselled regarding AD diagnosis. Enrolled in anti-amyloid therapy trial (donanemab).
Case 3: Patient in their 60s, former professional association football player with history of single mild TBI (car collision). ~5-year history of cognitive decline (memory, organisation, navigation), prominent personality change (disinhibition, obsessionality, mood swings, temper), reduced insight.	Severe memory impairment. Significant executive dysfunction (verbal fluency, Stroop, trails). Impaired processing speed. No affective symptoms.	Clinical MRI: mild small vessel disease. Frontal and anterior temporal atrophy with Sylvian fissure widening. Research DTI: Significantly reduced fractional anisotropy in the splenium of the corpus callosum.	Elevated NfL and S100B. Normal Aβ 42:40 ratio, p-tau181, total tau.	Probable behavioural variant frontotemporal dementia.	Signposted to specialist organisations for support (Rare Dementia Support, Alzheimer’s Society). Liaison with local memory team for ongoing behavioural management and support.
Case 4: Patient in their 50s, prior professional association football participation (defender, ~20-year career; ~5 symptomatic possible TBIs). Several years’ history of worsening memory (autobiographical, losing items), impacting work. Wife corroborates. Alcohol intake exceeds guidelines.	Variable memory performance (impaired on RBANS, better on WMS-IV); no definitive impairment pattern. No psychiatric symptoms.	Clinical MRI: Subtle right greater than left hippocampal atrophy. Non-specific white matter hyperintensities present, more than expected for age. Research DTI: Normal.	Isolated elevated NfL. Normal Aβ 42:40 ratio, p-tau181, total τ, S100B.	Possible early neurodegenerative process (non-AD) vs contribution from excessive alcohol use. Minimal objective cognitive impairment currently.	Counselled on diagnostic uncertainty, while noting the negative AD biomarkers and potential role of alcohol; advised to reduce intake. Planned follow-up with repeat neuropsychology and imaging.
Case 5: Patient in their 30 s, boxer (~15 years RHI exposure) with worsening irritability, impulsivity, violent outbursts and subjective memory complaints. History of prior contact with mental health services for ’stress'.	Variable memory scores (delayed>immediate recall). High self-reported executive issues (inhibition, emotional control). Mildly elevated depression/anxiety scores. Neuropsychiatric review remarkable for behaviour change with irritability, but without primary mental illness.	Clinical MRI: Normal structure. Research DTI: Normal.	Borderline elevated CSF NfL. Normal Aβ 42:40 ratio, p-tau181, total tau, S100B.	Neurobehavioural syndrome with subjective cognitive symptoms post-repetitive head impacts, with borderline raised NfL of uncertain significance.	Advised to cease boxing. Counselled in relation to diagnostic uncertainty, including the possibility of CTE pathology. Started low-dose risperidone for behavioural symptoms. For follow-up longitudinally,

All patients provided informed consent to participate in research (REC REF 17/LO/2066) and were assessed according a standardised protocol^78^ in the Advanced Brain Health Clinic/St Mary’s post-traumatic dementia clinic.

CTE: chronic traumatic encephalopathy.Aβ, amyloid-β; CSF, cerebrospinal fluid; DTI, diffusion tensor imaging; NfL, neurofilament light; p-tau181, phospho-tau181; RBANS, Repeatable Battery for the Assessment of Neuropsychological Status; RHI, repetitive head impacts; S100B, S100 calcium binding protein B; SD, standard deviation; TBI, traumatic brain injury; WMS-IV, Wechsler Memory Scale-Fourth Edition.

### What do former contact sports athletes present with?

Individuals with a history of repetitive head impacts commonly present with cognitive complaints, typically relating to memory, concentration and word-finding difficulties. In a midlife setting, these tend to be noticed by the patient and are less evident to family members or work colleagues. Impaired functioning in activities of daily living is relatively uncommon.^24^ Cognitive symptoms were common in our series of midlife rugby players, but no individual met the functional impairment criteria for dementia.^25^ Other common complaints include concerns about behaviour, particularly irritability or aggression, affective symptoms (anxiety >low mood) and headache. More rarely, patients complain of neurological symptoms such as dizziness or motor dysfunction. Family members often complain of behavioural issues, which may be associated with relationship difficulties. A relatively distinctive complaint is of memory impairments related to sporting fixtures in which the former player participated. This can occur in the absence of difficulties recalling recent events. If the patient sustained a TBI during the game, a period of post-traumatic amnesia may explain the lack of memory. More commonly, there is no clear history of TBI during the match, and it is unclear whether the lack of memory is abnormal. Many people are concerned that the inability to recall prior match fixtures indicates neurodegenerative dementia, yet there are several important differential diagnoses, including functional cognitive disorder^26^ (other differentials such as transient epileptic amnesia are far less likely and are associated with other distinctive clinical features).

One challenge in managing post-traumatic dementias is to identify syndromic clusters that map to post-traumatic pathologies. Historically, there have been attempts to define a syndrome of progressive and disabling cognitive and motor impairments associated with repetitive head impacts. This was first described in the late 1920s in Martland’s report of ‘punch-drunk’ boxers,^27^ and later refined by Critchley who termed this ‘chronic progressive traumatic encephalopathy’.^28 29^ More recent descriptions based on post-mortem findings in American footballers emphasised neuropsychiatric symptoms such as irritability and violent outbursts, with or without cognitive impairment, in an entity called ‘traumatic encephalopathy syndrome’, conceptualised to correspond to CTE.^25 30^ Unfortunately, the traumatic encephalopathy syndrome *research* diagnostic criteria are not yet ready for use in clinical practice, being formulated across several US brain bank case series reflecting predominantly American football exposures, with uncertain sensitivity/specificity in other sports.

### Which neuropathologies might be present?

**Figure 1:**
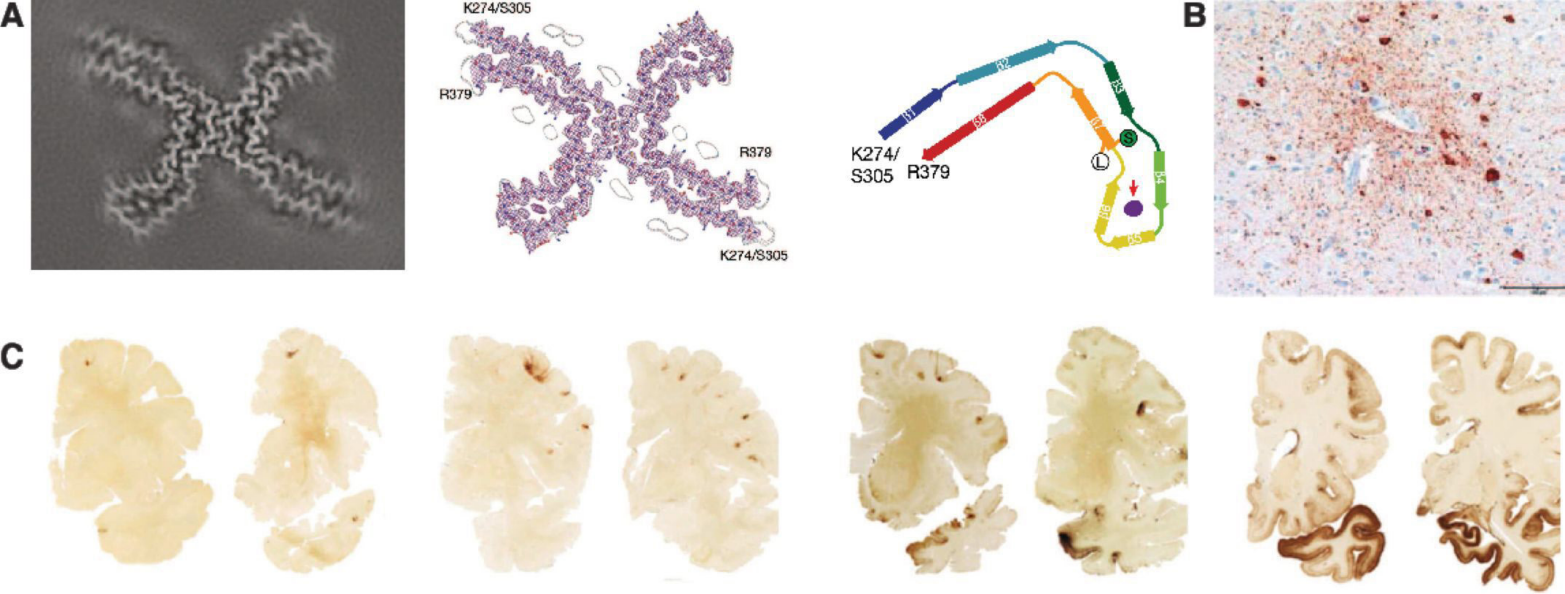
Chronic traumatic encephalopathy (CTE) neuropathology and staging. (**A**) Distinctive τ structure in CTE. Cryo-electron microscopy (cryo-EM) structure of CTE type I tau filament from temporal cortex of a former American footballer (left). Sharpened high-resolution cryo-EM map overlaid with atomic model (middle). Schematic of the secondary structure elements in the CTE fold, with an extra density of ‘unknown identity’ not seen in AD tau, shown in purple and with a red arrow shown (right); images adapted from Falcon *et al* (ref 6055370738038).^31^ (**B**) Perivascular phospho-tau (AT8) positive neurofibrillary tangles and neurites at the cortical sulcal depth, the ‘pathognomonic CTE lesion’ in a former American footballer; adapted from Bieniek *et al* (ref 6062130177069).^35^ (**C**) McKee CTE staging (using AT8 p-tau stain), comprising stages I–IV shown from left to right, with increasing spatial involvement of tau pathology. Stage I: focal perivascular lesions (frontal cortex). Stage II: multiple cortical foci, early subcortical spread. Stage III: widespread cortical and mesial temporal involvement, brainstem pathology and gross atrophy. Stage IV: severe, extensive pathology across multiple brain regions with marked neurodegeneration and frequent co-pathologies such as TAR DNA-binding protein 43 (TDP-43). Adapted from McKee *et al* (ref 6062130399007).^79^

CTE is a progressive mixed 3/4-repeat tauopathy characterised by paired helical filament neurofibrillary tau tangles.^31^ The neuropathology was initially characterised in detail by Nick Corsellis at Runwell Hospital in Essex. In a post-mortem series of former boxers with dementia, he described neurofibrillary tangles in a distribution distinct from AD, with a relative absence of senile plaques (ie, Aβ). More recently, Ann McKee described CTE pathology in American footballers,^32^ where intraneuronal (predominantly 3-repeat) tau pathology has a characteristic distribution around blood vessels particularly in the cortical sulcal depths (see figure 1)^33^ with 4-repeat tau also seen predominantly in astrocytes.^34 35^ Modern analyses of the Corsellis collection at Imperial College London, have confirmed the presence of CTE in a substantial proportion of cases using modern pathological diagnostic criteria.^36^ Electron microscopy has recently shown the structure of paired helical filaments in CTE tau to be distinct from AD.^31^ Intriguingly, it is identical to that of subacute sclerosing panencephalitis, a progressive cognitive impairment seen occasionally after measles virus exposure, as well as the ALS/Parkinson’s dementia complex and vacuolar dementia (figure 1).^37 38^

There are four stages of CTE (figure 1C) based on the hyperphosphorylated tau (p-tau) distribution seen post mortem. Levels of cognitive impairment and functional limitation increase with stage, with relatively low dementia rates in stage I (~27%, mean age 45 years) increasing in stage IV (93% with dementia, mean age 76).^39^ A longer playing career is predictive of CTE p-tau burden in brain bank data,^40^ though there have been reported cases in young people and amateurs with a lower cumulative dose.^41^ Using biomechanical data such as using helmet sensors improves CTE risk estimates.^42^

As in many neurodegenerative contexts, co-pathologies are very common and may contribute differentially to the phenotype. In a large brain bank series of patients with repetitive head impacts, most patients (60%) had two or more neuropathologies,^23^ and the presence of a specific pathology does not indicate that this is the primary driver of symptoms. In one case series,^23^ Alzheimer’s pathology explained far more of the cognitive impairment experienced by patients than did CTE (more than twice the amount), despite being less common, highlighting the need to consider multiple potential contributors to the clinical syndrome.

It is very challenging to comment on the population prevalence of post-traumatic pathologies as we have no reliable non-invasive biomarkers for most pathologies in life. Many retired athletes consulting neurologists will not have neurodegenerative (or TBI) pathology, and neurologists should note that much of our current knowledge comes from limited brain bank data, which are likely to be poorly representative of the wider population.

### History taking

Cognitive symptoms should be elicited in history taking. These include subjective difficulties with memory, attention, executive function and speed of processing. Complaints of attentional lapses and difficulties with organisation and/or planning are common. Specific questions about salient recent events can help in assessing memory function. For example, we often ask retired players about details of recent matches they have watched, which can be very discriminating as an objective measure of memory impairment. Symptoms of dysfunction in other domains such as language, calculation, praxis and visuospatial function should be evaluated. It is often initially unclear whether these impairments are progressive or static in nature. This is particularly difficult to determine after a single assessment. Collateral history and repeated assessment are needed to clarify whether problems are progressive. The degree of functional impairment in instrumental activities of daily living should be assessed and documented.

Neurobehavioural dysregulation symptoms such as explosiveness, impulsivity, rage, violent outbursts and emotional lability should be assessed, including any forensic issues. These may occur in isolation from cognitive impairment and are described as a ‘core feature’ of traumatic encephalopathy syndrome, with some association with frontal CTE pathology.^43^ Affective problems, particularly depression and anxiety symptoms, are common and can contribute to neurological complaints. We use standardised questionnaires (Beck Depression Inventory 2nd Edition - BDI-II and the Hospital Anxiety and Depression Scale - HADS) to screen for clinically relevant symptoms, but patients often need psychiatric assessment to clarify the diagnosis. Psychosis is uncommon, except in the context of post-traumatic amnesia (acutely after injury), in advanced neurodegenerative disease or in the presence of drug abuse. Post-traumatic stress disorder is also uncommon after repeated head impact exposure in a sporting context, but may be more common in military settings,^44^ particularly when memory for the index injury is retained. Psychiatric problems that pre-date TBI are a vulnerability factor for poor outcomes,^45^ and the presence of a pre-existing mental health condition makes it more likely that patients will develop a psychiatric disorder after head injury.^46^ It is useful to enquire about symptoms relevant to key differential diagnoses, particularly behavioural variant frontotemporal dementia, such as obsessionality, new fixed rituals, loss of empathy or social awareness (eg, inappropriate humour, sexual disinhibition), gambling and changes in dietary preference (eg, developing a sweet tooth). A dedicated neuropsychiatric assessment may help, particularly where symptoms are causing problems for patients and/or family members. The causation of neuropsychiatric symptoms is complex in this setting. Clinically, it may be difficult to differentiate primary psychiatric problems from psychiatric symptoms arising due to an early-stage neurodegenerative problem: here, the clinical context is important (eg, are there associated cognitive problems, abnormal examination findings?), as is evaluating the patient over time for progression.

Clinicians should obtain a detailed history of repetitive head impacts and any associated TBI exposure. The risk of late problems appears to relate to cumulative head trauma load.^42^ Players usually present after many years of exposure, so a precise individual estimate of exposure is not possible. However, the duration of playing career, type of involvement (professional, amateur and/or youth), the positions played and the number of concussions sustained provide useful proxies for the cumulative exposure. For American football participation, more precise estimates of head impact exposures for specific player positions have been generated in the research setting,^42^ but these are not available for sports commonly played in the UK. In the future, much more information about cumulative head impact exposure will be available for some players: most professional rugby players in the UK now wear instrumented mouth guards.^47^ Hence, in the future, some players will present to clinic with precise information about their cumulative exposure, which may prove useful in evaluating long-term risk.

The assessment and management of concussion has been an important focus of sports medicine and sporting governing bodies in recent years.^48 49^ As we have previously argued,^10^ using concussion as a diagnosis or as the focus for clinical management is problematic because the heterogeneous symptoms that comprise concussion or post-concussion syndrome have varying pathophysiology. However, players are almost always familiar with the term and can provide information about symptomatic head impacts during their careers that they may have considered concussions, although there is a wide variability in the way concussions are reported retrospectively. Our pragmatic approach is to document the number of concussions a player reports using standardised tools such as the Mayo classification of TBI^50^ (see table 2 for clinical and imaging features which define different severity/confidence levels), but we avoid using concussion or post-concussion syndrome as diagnostic labels. For symptomatic head impacts, loss of consciousness, retrograde or post-traumatic amnesia duration, hospitalisation, imaging findings and recovery time (including return-to-play protocols) should be documented. We recommend using the widely used Mayo classification of TBI severity,^49^ which incorporates clinical features such as the Glasgow coma scale and post-traumatic amnesia duration, but also makes use of neuroimaging findings, classifying each injury as symptomatic possible, mild probable, moderate-severe^50^ (table 2).

**Table 2:** Mayo classification of traumatic brain injury

**Symptomatic (possible**) if does not meet criteria for a higher classification, and if one or more of the following are present:	**Mild (probable**) if does not meet criteria for a higher classification, and if one or more of the following apply:	**Moderate-severe (definite**) one or more of the following apply:
**Clinical features**	Blurred vision Confusion (mental state changes) Dazed/dizziness Focal neurological symptoms Headache Nausea	Loss of consciousness momentary to <30 min Post-traumatic anterograde amnesia momentary to <24 hours Depressed, basilar or linear skull fracture (dura intact)	Death due to TBI Loss of consciousness ≥30 min PTA ≥24 hours Worst Glasgow Coma Scale full score in first 24 hours 13 (unless invalidated on review, eg, attributable to intoxication, sedation, systemic shock)
**Neuroimaging**	NA	NA	Contusion or intracerebral haematoma Subdural haematoma Extradural haematoma Penetrating TBI (dura penetrated) Subarachnoid haemorrhage Brainstem injury

Adapted from Malec *et al*.^50^

PTA, post traumatic amnesia; TBI, traumatic brain injury.

Systemic health issues relevant to brain health should be screened for. We pay particular attention to the management of cardiovascular risk factors (eg, smoking, lack of exercise, hypertension, hyperlipidaemia and hyperglycaemia). Drug side effects can be relevant, including risks from anticholinergics, opiates and sedatives. Excess alcohol consumption and recreational drug use should be assessed. In our experience, sleep abnormalities are common, resulting from a range of factors including psychiatric problems, chronic pain, obstructive sleep apnoea and neurodegenerative conditions. Retired players often have complicated orthopaedic histories and may have chronic pain, which can influence other aspects of the presentation.

A collateral history should also be obtained. Informants should be asked to comment on current problems, how they have evolved over time and whether the problems are progressive. It is useful to explore their views about their social interactions, vocational difficulties, self-awareness and mental state. From a neurodegenerative perspective, reports of progressive impairments that are substantiated in the collateral history are important, particularly if there was a clear delay in the onset following the cessation of repetitive head impacts/TBI exposure. It is also important to clarify the family history and sensitively to enquire whether the patient is involved in any compensation claims or legal action related to their health, as this can be a source of stress and may involve external assessments that are relevant to the diagnostic workup.

## Examination

Although objective neurological signs are rarely identified in patients under 60, a full neurological examination is important. Eye movements should be carefully assessed, alongside looking for features of Parkinsonism, ataxia, dysarthria, imbalance and testing for evidence of motor neurone disease such as fasciculation. These features can help to refine the phenotype and have all been reported after sporting exposures. In our experience, unusual combinations of signs can be present in the post-traumatic setting. This is particularly true in more severe cases of TBI where complex movement disorders sometimes occur. Cognitive function should be objectively assessed and schema such as the ‘walk around the brain’ may assist the neurologist, comprising features of behaviour, language, reading, spelling, calculation, working/episodic/topographical memory, praxis, object analysis, spatial awareness and perception (as recently described in detail in practical neurology).^51^ Screening tests such as the Montreal cognitive assessment (MoCA),^52^ Mini-mental state examination (MMSE)^53^ or Addenbrooke’s cognitive assessment (ACE-III)^54^ have limitations, but provide a pragmatic approach to initial assessment and can help in tracking change over time and provide a high-level indication of cognitive performance at that moment. Our preference is to use the ACE-III for screening of cognitive impairments associated with either long-term effects of repetitive head impacts or TBI and/or neurodegenerative effects.

## Investigations

Investigations are needed to assess cognitive function objectively, identify evidence of TBI and/or progressive neurodegeneration, exclude mimics and identify important co-pathologies. The classification of neurodegenerative conditions is moving towards a multidimensional approach that reflects the underlying neurobiology of the conditions (eg, the amyloid/tau/neurodegeneration framework for AD).^55^ In a similar way, the diagnosis of TBI and the evaluation of its consequences is moving toward a classification based on the neurobiology of the initial insult and the identification of pathophysiology triggered by this injury. The National Institute of Neurological Disorders and Stroke recently proposed a new classification of TBI comprising clinical features, biomarkers, imaging and modifiers. As TBI blood biomarkers complete their validation and are available for routine clinical use, we expect these approaches will become mainstream.^56^

### Objective assessment of cognitive function

Formal neuropsychological testing is recommended when there are significant concerns about cognitive function identified from the history and screening assessments. This provides objective assessment, which is particularly important as it is often discrepant from subjective concerns. Assessments will also clarify the patterns of deficits and establish a baseline for monitoring potential progression. Key aspects to assess are an estimate of premorbid cognitive functioning, memory, processing speed, visuospatial functioning, language, working memory, attention and executive functioning. Performance validity testing should be included, particularly in the context of personal injury claims. There are limits with the ability of executive tests to detect executive difficulties and the presence of behavioural problems on patient report or collateral history providing clues to the presence of executive impairments. Our approach is to use a formal assessment of the dysexecutive behaviour completed by the patient, and an informant (Behavior Rating Inventory of Executive Function-A,^57^ the Frontal Systems Behaviour Scale^58^ and the Dysexecutive Questionnaire)^59^. Collateral information from an informant is essential, particularly in individuals who may have reduced insight into their everyday cognitive difficulties. Single neuropsychology assessments can be misleading. In our experience, variability in performance (both improvement and declines) is common over a 2-year period. Hence, neuropsychology findings should be viewed in the wider clinical context and not over-interpreted.

### Neuroimaging

Prior CT scans should be reviewed as these may show the acute or late effects of TBI (eg, contusions, subdurals, extradurals, post-injury gliosis) and neurodegeneration (ie, brain atrophy). One potentially relevant abnormality is cavum septum pellucidum, where the interventricular septum pellucidum is separated with an enlarged cavity centrally, sometimes with fenestration of the leaflets. While a small cavum septum pellucidum (a triangular opening at the anterior horns of the lateral ventricles) is considered to be non-specific and can frequently occur without a history of injury,^60^ an ‘enlarged’ cavum septum pellucidum (ie, radiological grade 2 or larger)^61^ is more common after repetitive head impacts and TBI exposure, where it is associated with poorer memory performance.^62^

**Figure 2:**
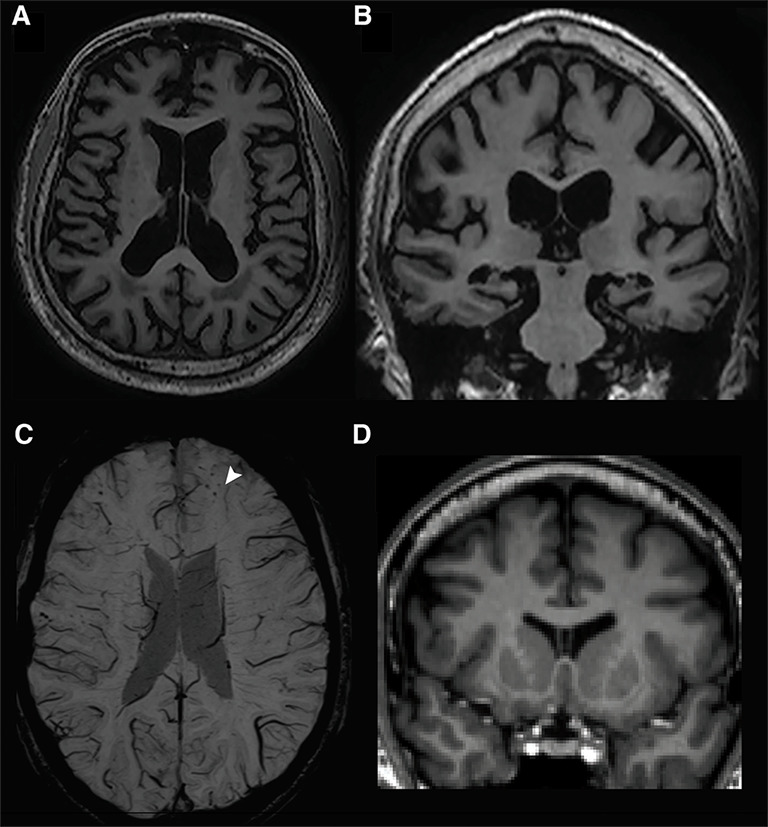
Neuroimaging features in former contact sports athletes. Volumetric T1 MR scan of brain in a patient in their 80s with history of American football participation and clinical syndrome of dementia, with CTE present at post-mortem. Axial (**A**) and coronal (**B**) views showing prominent frontotemporal atrophy and cavum septum pellucidum posteriorly; images adapted with permission from Alosco *et al.*^39^ (**C**) Axial SWI MRI in midlife former rugby player showing left frontal microbleeds (white arrowhead). (**D**) Cavum septum pellucidum shown on coronal T1 MRI in former rugby player. CTE, chronic traumatic encephalopathy; SWI, susceptibility-weighted imaging.

MR imaging is informative in assessing both TBI and neurodegenerative pathology. The assessment should include volumetric T1-weighted sequences (for focal TBI and atrophy), T2-FLAIR (for white matter hyperintensities suggesting vascular disease or gliosis) and susceptibility-weighted imaging (SWI, to assess for microhaemorrhages). Focal traumatic injuries may be identified on T1/FLAIR and volumetric T1 atrophy is a non-specific marker of neurodegeneration. Patterns associated with CTE include relative prominence in orbitofrontal, dorsolateral frontal and anterior/medial temporal regions, often with ventricular enlargement (figure 2A), which may correlate with τ burden.^63^ SWI can identify microhaemorrhages consistent with past traumatic vascular injury, potentially related to traumatic axonal injury,^64^ hypertensive disease or cerebral amyloid angiopathy. Post-traumatic microhaemorrhages often occur in a parafalcine distribution, which can help in distinguishing them from other causes. Diffusion tensor imaging (DTI) is an MRI technique sensitive to white matter microstructure widely used in research^64^ but which for technical reasons has been challenging to translate into widespread clinical use. DTI can be used to identify white matter abnormalities associated with diffuse axonal injury at the individual level.^64^ DTI white matter abnormalities were seen in 17% of active professional rugby players,^65^ although similar abnormalities were observed in ~5% of retired professional rugby players.^66^ Hence, unlike moderate to severe TBI, it is unclear whether DTI has a role in individual patient diagnosis or management in the chronic phase after repetitive head impacts, and there is no current evidence that DTI changes correlate with specific neuropathologies like CTE at the individual level.

The role for positron emission tomography (PET) in a clinical setting is limited in this context. [18F]FDG-PET, assessing cerebral metabolism, lacks clear evidence of utility in CTE, although it may provide non-specific information. Amyloid PET (eg,[18F]Florbetapir, [18F]Florbetaben) can help to confirm or exclude significant amyloid plaque burden (consistent with AD) if CSF analysis is not possible or desired, although widespread availability of plasma Alzheimer’s biomarkers may reduce its necessity. The tau PET ligand 18-F flortaucipir has MHRA approval to support the diagnosis of AD, but at present, neither this tracer nor a range of other tau PET ligands has yet shown satisfactory sensitivity/specificity for CTE tau.^67^

Functional neuroimaging techniques such as fMRI may reveal abnormalities acutely following TBI;^68^ however, there is not yet any clear evidence of clinical utility in assessing former athletes presenting with cognitive problems. We need more investigation of these tools, including related techniques such as magnetoencephalography (MEG).

### Blood and CSF analyses

Initial investigations should include standard blood workup to rule out reversible or contributing factors. This may include full blood count, renal profile, liver function test (including gamma-GT), bone profile, C-reactive protein, ESR (erythrocyte sedimentation rate), vitamin B12, folate, HIV, VDRL (Venereal Disease Research Laboratory assay), HbA1c (glycosylated haemoglobin) and thyroid function.

Fluid biomarkers of neurodegeneration and neuropathology, primarily from CSF, can help to establish whether neurodegeneration is present (eg, via neurofilament light or total tau) and whether AD pathologies are present (eg, amyloid beta 42:40 ratio, p-tau_181_, p-tau_217_; with the latter two thought to reflect both amyloid and tau pathology). Phospho-tau fluid biomarkers are not thought to be raised in cases of CTE without AD co-pathology,^69^ though they can usefully determine the presence of AD (co-)pathology, informing decisions about treatments approved for Alzheimer’s.

Ultrasensitive assays mean that biomarkers can, in a research setting, be reliably quantified at low quantifications in blood. Blood p-tau217 has particularly strong sensitivity/specificity for AD pathology,^70^ while we and others have validated fluid biomarkers for TBI (glial fibrillar acidic protein, neurofilament light, total tau), cerebral Aβ pathology (plasma Aβ42/40 ratio) and tau/amyloid pathology (P-tau_181_ and _217_) for use in TBI studies in the acute/subacute and chronic phases.^71 72^ In our Institute of Sport, Exercise and Health clinic, we have seen elevations of p-tau_217_ in around 25% of retired rugby players assessed, which may indicate the presence of amyloid dependent tau pathology, although further work is needed to clarify the clinical interpretation and implications for diagnostic assessments.^66^

### Genetics

Genetic factors play a role in determining outcomes after TBI and are likely to influence the risk of neurodegeneration. However, the studies performed to date are likely to be too small to clarify this issue reliably and have largely focused on genetic influences on TBI clinical outcomes rather than the genetic influences on neurodegenerative risk after TBI. From a practical perspective, genetic investigations are particularly useful in cases where frontotemporal dementia is high in the differential diagnosis, including due to mutations such as *C9ORF72*, which can produce a wide phenotype including features of motor neurone disease. In addition, amyloid precursor protein and presenilin 1/2 mutations may help in evaluating Alzheimer’s presentations, as reviewed in *Practical Neurology* recently.^73^ APOE (apolipoprotein E) has been reported to be a risk factor for poor clinical outcome after TBI, as well as its role in Alzheimer’s risk. However, we do not currently test APOE clinically in our brain health clinic, although this is adopted in some centres^74^ with appropriate patient counselling to inform risk of AD. Moving forward, this may assist in determining eligibility for anti-amyloid treatments, as e4 homozygosity is considered a contraindication due to increased risk of amyloid related imaging abnormalities. Patients sometimes present with the effects of genetic disease, which they falsely attribute to the effects of head impact exposure or TBI (eg, one case of CANVAS presenting with slowly progressive cerebellar syndrome in a retired rugby player—see recent *Practical Neurology* review for discussion of this issue).^75^

## Management

### Diagnostic formulation

We take a stepwise approach to the following questions: (i) has there been substantial head impact/TBI exposure, and is there evidence of TBI clinically; (ii) is there cognitive impairment and/or neuropsychiatric symptoms; (iii) is there a progressive worsening and/or impairment of daily function; (iv) are criteria for a canonical neurodegenerative disease such as AD, frontotemporal dementia or dementia with Lewy bodies met; (v) is there a non-neurodegenerative cause for the presentation such as psychiatric morbidity or sleep disorder? A cautious approach is merited in attributing the symptoms to repetitive head impacts, TBI or neurodegeneration, particularly given the prognostic implications of such a diagnosis. The neurologist needs to consider the strong priors for non-neurodegenerative explanations of many symptoms in young adults, while recognising that neurodegenerative disease rates increase markedly with age.

Patients with significant concerns are best assessed in a multidisciplinary clinic, ideally with input from neurology, neuropsychiatry and neuropsychology. Consensus assessment is helpful given the prominent mixtures of cognitive, neurological and psychiatric symptomatology. Initial lighter touch screening of patients with concerns about brain health given a history of head impact exposure is a pragmatic approach to dealing with large patient numbers and avoids unnecessary investigations.

We avoid using the term traumatic encephalopathy syndrome in routine clinical diagnosis as it is an unvalidated research construct, with uncertain accuracy especially outside American football players. People often ask directly if their symptoms are due to their sporting career. It is important to acknowledge that head trauma exposure is a dementia risk factor, but attributing an individual’s symptoms definitively to repetitive head impacts is often challenging due to the multifactorial nature of dementia and current diagnostic limitations. Explaining this uncertainty is key. For individuals still actively exposed to repetitive head impacts, counselling about the risks of continuing should involve a discussion of these uncertainties, consideration of any existing clinical impairment or imaging abnormalities and shared decision-making regarding strategies to minimise further exposure versus personal preferences. We have found that making clear diagnoses can be therapeutically helpful, particularly when there is anxiety associated with the possible presence of neurodegenerative disease.

### Medications and non-pharmacological support

Management focuses on symptomatic treatment and promoting brain health. Conditions such as depression, anxiety and sleep disorders are common and can significantly impact cognition. Targeted investigation and treatment, including psychological therapies and psychiatric medications, are often beneficial and should be prioritised, particularly in those with subjective complaints but no clear neurodegenerative process. Neurobehavioural dysregulation symptoms such as irritability and agitation may respond to selective serotonin reuptake inhibitors in the first instance, or low-dose atypical anti-psychotics such as risperidone should these prove ineffective.

In people with dementia and evidence of Alzheimer’s pathology, if there is no contraindication, our practice is to use cholinesterase inhibitors (eg, donepezil, galantamine or rivastigmine), or a N-methyl-D-aspartate (NMDA) antagonist such as memantine. However, there is no specific evidence to support this in the setting of cognitive impairment following repetitive head impacts thought to be due to CTE without Alzheimer’s pathology. In contrast to TBI, where stimulants such as lisdexamfetamine and methylphenidate^76^ may be helpful off-label for attentional problems, there is no evidence to support their use after repetitive head impacts.

Clinicians should take the opportunity to provide individualised advice for maintaining life-long brain health. This involves optimising vascular risk factors (blood pressure, cholesterol, diabetes control), encouraging physical activity and socialisation and addressing other factors such as sensory impairment.^77^

Referral to community memory services is important for ongoing support in people who have progressive problems, particularly for the management of behavioural and psychiatric symptoms. Practical support is available from third-sector organisations. Dementia UK’s Admiral Nurse Dementia Helpline supports a dedicated nurse specialising in dementia after sports participation, offering free advice and support (patients can self-refer via dementiauk.org/get-support/dementia-helpline-information/). Former professional footballers can access specific resources via the Professional Footballers’ Association Brain Health team (thepfa.com/players/brain-health). The Alzheimer’s Society also provides valuable resources and support, applicable beyond just AD (alzheimers.org.uk).

## Future directions

Improving diagnosis and care requires further research. Several UK studies are active, such as the ABHC study investigating brain health in former elite rugby players and association footballers.^78^ Clinicopathological correlation across diverse sporting exposures is key to refining diagnostic criteria and understanding risk factors. Given our current inability to definitively diagnose CTE during life, discussing the possibility of brain donation for research is particularly important. Several UK brain banks focus specifically on the sequelae of trauma and repetitive head impacts, including the Medical Research Council (MRC) Brain Bank at the University of Oxford (linked with the Concussion Legacy Foundation UK) and the Glasgow Brain Injury Research Group. Clinicians can facilitate contact if patients express interest.

**Further reading**

- Parker T, Hain JA, Rooney EJ, et al. Brain Health Concerns in Former Rugby Players: Clinical and Cognitive Phenotypes. Brain. 2025;doi:10.1093/brain/awae416
- Graham NS, Zimmerman KA, Hain JA, et al. Biomarker evidence of neurodegeneration in midlife former rugby players. Brain. 2025; 10.1093/brain/awaf152
- Saltiel N, Tripodis Y, Menzin T, et al. Relative contributions of mixed pathologies to cognitive and functional symptoms in brain donors exposed to repetitive head impacts. Ann Neurol. Nov 3 2023;doi:10.1002/ana.26823

**Key points**

- Retired players may develop neurological or psychiatric problems that relate to previous injuries, psychological factors or, more rarely (in those <60 years), neurodegeneration.
- Substantial repetitive head impacts exposure and single traumatic brain injury are associated with increased dementia risk, so clinicians should systematically take a head injury history.
- However, many cognitive complaints are explained by treatable non-neurodegenerative issues such as psychological/psychiatric factors, sleep or pain.
- Features suggesting underlying neurodegeneration include caregiver concern, progressive functional decline, abnormal examination, objective cognitive deficits and supportive biomarkers (which remain non-specific for chronic traumatic encephalopathy).
- Ex-player brain health should also be supported by addressing modifiable dementia risk factors such as smoking, alcohol, blood pressure management, obesity, physical activity, hyperglycaemia, hyperlipidaemia, low mood, isolation and sensory impairment.

## Data Availability

No data are available.
